# Molecular Epidemiology, Lineage Evolutionary Dynamics, and Antigenic Variation Analysis of Type II PRRSV in China During 2024–2025

**DOI:** 10.1155/tbed/2054759

**Published:** 2025-09-27

**Authors:** Dihua Zhu, Guangyu Liu, Huixin Li, Fen Li, Xiaolong Xu, Yuanyuan Fu, Pandan Chen, Guihong Zhang, Yankuo Sun

**Affiliations:** ^1^Guangdong Provincial Key Laboratory of Zoonosis Prevention and Control, College of Veterinary Medicine, South China Agricultural University, Guangzhou 510642, China; ^2^Maoming Branch, Guangdong Laboratory for Lingnan Modern Agriculture, Maoming 525000, China; ^3^National Engineering Research Center for Breeding Swine Industry, South China Agricultural University, Guangzhou 510642, China; ^4^Animal Husbandry and Veterinary Service Center of Ledong Li Autonomous County, Hainan Province, China

**Keywords:** antigenic variation, gene recombination, intrahost single nucleotide variant, lineage evolution, molecular epidemiology, porcine reproductive and respiratory syndrome virus, transmission dynamics

## Abstract

Porcine reproductive and respiratory syndrome virus (PRRSV) represents one of the major threats to the global swine industry, with its rapid evolution and antigenic variation posing persistent challenges to disease control. Based on 328 clinical samples collected from PRRSV symptomatic (respiratory disorders, reproductive failures, and high fever) pigs across 27 provinces in China during 2024–2025, this study employed open reading frame (ORF) 5 gene sequencing, complete genome sequencing of representative strains from key lineages (including a rapidly spreading NADC30-like Lineage 1.8 strain, a vaccine-related Lineage 8.7 strain, and a genetically distinct Lineage 3 strain), phylogenetic analysis, transmission dynamics analysis, intrahost single nucleotide variant (iSNV) analysis, and recombination detection to systematically reveal the molecular epidemiological characteristics and evolutionary dynamics of type II PRRSV currently circulating in China. The results demonstrated a complex pattern of coexistence among five major lineages of type II PRRSV in China, including Lineage 1.5, Lineage 1.8, Lineage 3, Lineage 5, and Lineage 8.7, with Lineage 1.8 emerging as the predominant circulating strain (48.5% of positive samples), followed by Lineage 1.5 (23.2%), while Lineages 3, 5, and 8.7 showed more restricted geographical distribution. Spatial transmission analysis identified Guangdong and Henan as key transmission nodes, forming “viral exchange centers” connecting northern and southern regions, while Hubei, Shanxi, and Jiangsu have become new viral aggregation sites. Genetic diversity analysis revealed high haplotype diversity (Hd) across all lineages except Lineage 5, with Lineage 5 showing a remarkable 106.6% increase in nucleotide diversity within 1 year, indicating rapid adaptive evolution. Tajima's D test results revealed negative values for most lineages, with Lineage 5 and 8.7 reaching statistical significance, suggesting these viral populations have undergone recent population expansion or directional selection. Multidimensional scaling (MDS) analysis based on genetic distance revealed a potential antigenic divergence between the predominant circulating lineages (1.8 and 3) and current vaccine strains, which may compromise vaccine efficacy. In-depth analysis of three representative genomes revealed complex recombination patterns involving vaccine-related strains and identified the ORF2-ORF3 region as a potential recombination hotspot. The findings of this study provide a scientific basis for understanding the evolutionary mechanisms of type II PRRSV in China and offer important references for formulating targeted control strategies and optimizing vaccine design, which has significant value for ensuring the healthy development of China's swine industry in the post-African swine fever era.

## 1. Introduction

Porcine reproductive and respiratory syndrome (PRRS), caused by the PRRS virus (PRRSV), is a highly contagious disease that has become one of the most economically devastating pathogens in the global swine industry since its first identification in the United States in 1987 [1, 2]. As the world's largest pork producer and consumer, China's swine industry is particularly vulnerable to PRRSV circulation. The continuous prevalence of North American (type II) PRRSV has posed sustained challenges to swine health and industry economics in China. Since its initial introduction to China in 1995, type II PRRSV has undergone complex evolutionary processes. In 2006, the emergence of highly pathogenic PRRSV (HP-PRRSV) caused catastrophic outbreaks, resulting in millions of pig deaths and enormous economic losses [3]. However, in recent years, Lineage 1 variants represented by NADC30-like and NADC34-like sublineages have gradually replaced HP-PRRSV to become the new dominant circulating lineages in China [4].

PRRSV exhibits remarkable genetic diversity and antigenic variation capabilities, closely related to its nature as an RNA virus. The PRRSV genome is approximately 15 kb in length and contains approximately 10 open reading frames (ORFs). Among these, nonstructural protein 2 (nsp2) and the major envelope glycoprotein (GP5, encoded by ORF5) regions exhibit the highest mutation rates and are therefore widely used in molecular epidemiology studies [5]. However, systematic research on the mechanisms of PRRSV lineage dynamic evolution in Chinese swine herds and its impact on vaccine efficacy remains limited, particularly in the post-African swine fever era, where new challenges continuously emerge as the swine industry rapidly recovers and rebuilds [6].

Notably, during 2024–2025, type II PRRSV in China has demonstrated new epidemiological characteristics. Compared to previous periods, not only has viral lineage diversity increased, but recombination events and antigenic drift phenomena have also significantly intensified. The rapid expansion and trans-regional spread of NADC34-like lineages, in particular, has attracted widespread attention from the industry [7]. More concerningly, recombination events between vaccine strains used in the market (such as JXA1, CH-1R, and MLV) and wild type strains have been increasingly reported, potentially affecting vaccine safety and efficacy and possibly generating recombinant viruses with novel antigenic properties or enhanced virulence [8].However, to date, systematic studies on the molecular epidemiological characteristics, lineage diversity, and antigenic variation of type II PRRSV in China during 2024–2025 remain scarce. Comprehensive in-depth analyses are particularly lacking regarding genetic variation patterns of emerging lineages, recombination hotspots, and mechanisms of antigenic drift.

To address this research gap, our study utilized comprehensive methods, including whole-genome sequencing, phylogenetic analysis, recombination detection, and antigenic variation analysis based on 328 samples collected from 27 provinces in China during 2024–2025. This represents the first systematic investigation of the spatiotemporal dynamics, evolutionary patterns, and antigenic variation characteristics of type II PRRSV in China during this period. We discovered the coexistence of major lineages in China, with Lineage 1.8 being predominant, and identified two key transmission nodes functioning as “viral exchange centers” connecting northern and southern regions. Our antigenic analysis further revealed antigenic divergence between current vaccine strains and the predominant circulating lineages, which may compromise vaccine efficacy. Through this study, we aim to investigate the distinct intrahost single nucleotide variant (iSNV) patterns among different lineages, particularly in antigen-related regions, identify critical recombination hotspots, and assess the antigenic match between existing vaccine strains and circulating field strains. This research not only addresses a significant gap in molecular epidemiological studies of PRRSV in China but also provides a scientific foundation for developing targeted control strategies and optimizing vaccine design. Furthermore, our findings will contribute to elucidating the evolutionary mechanisms and antigenic variation patterns of PRRSV in Chinese swine herds, offering new perspectives on viral adaptive evolution and immune escape. These insights hold profound implications for the sustainable development of China's swine industry in the post-African swine fever era, the improvement of disease prevention and control systems, and the enhancement of biosecurity levels. Ultimately, this work will provide crucial support for safeguarding national food security and promoting the healthy development of the pork production sector, which is of paramount importance for both economic stability and public health.

## 2. Materials and Methods

### 2.1. Sample Collection

Between 2024 and 2025, a total of 328 clinical samples were collected from pigs exhibiting typical symptoms of PRRS across 27 provinces in China. Specimens, including serum, lung tissue, tonsils, and lymph nodes, were collected under aseptic conditions and transported to the laboratory at 4°C for subsequent processing.

### 2.2. ORF5 Gene Amplification and Sequencing

Viral nucleic acids were extracted using the Vazyme Virus DNA/RNA Extraction Kit 2.0 (Nanjing Vazyme Biotech Co., Ltd., China) according to the manufacturer's instructions. The ORF5 gene fragment was amplified by RT-PCR using specific primers PRRSV-GP5-F (5′-GTTTACCCAACGCTCCTTA-3′) and PRRSV-GP5-R (5′-ACTGGCGTGTAGGTAATGG-3′), a primer set that has been previously described and used for ORF5 amplification [9]. The PCR reaction mixture (50 μL) contained: 25 μL of 2x Phanta Max Master Mix (Vazyme), 1 μL each of forward and reverse primers (10 μM), 5 μL of template nucleic acid, and 18 μL of deionized water. The PCR thermal cycling conditions were as follows: initial denaturation at 98°C for 3 min; 35 cycles of denaturation at 98°C for 15 s, annealing at 58°C for 30 s, extension at 72°C for 45 s; and a final extension at 72°C for 10 min. After verification by 1.5% agarose gel electrophoresis, the target bands were excised, purified, and subjected to bidirectional Sanger sequencing using an ABI 3730XL automated sequencer (Applied Biosystems, USA) to obtain complete ORF5 gene sequences.

### 2.3. Complete Genome Sequencing and Data Processing

To further investigate the evolutionary dynamics, recombination patterns, and antigenic variation of the major PRRSV lineages, we selected three representative strains from Lineages 1.8, 3, and 8.7 for complete-genome sequencing. These strains were chosen based on their distinct phylogenetic positions and epidemiological relevance. Sequencing libraries were constructed using the VAHTS Universal V8 RNA-seq Library Prep Kit for MGI. The extracted RNA was fragmented and reverse-transcribed into first-strand cDNA using random primers, followed by second-strand cDNA synthesis, double-stranded cDNA end repair, dA-tailing, adapter ligation, dual-round bead-based size selection, PCR enrichment, and library purification. The purified libraries were analyzed using Qsep100 for fragment size distribution assessment and quantified using Qubit for double-stranded DNA concentration measurement. Qualified libraries were sequenced on the MGISEQ-200 sequencer on the DNBSEQ-T7 platform. Raw sequencing data underwent quality control using FastQC (https://www.bioinformatics.babraham.ac.uk/projects/fastqc/) and quality trimming using Trimmomatic (parameters: LEADING:3 TRAILING:3 SLIDINGWINDOW: 4:15 MINLEN:36) to remove low-quality sequences and adapter contamination [10]. The processed reads were de novo assembled using MEGAHIT (parameter:—min-contig-len 200), and the resulting contigs were identified as PRRSV sequences through BLAST comparison against the PRRSV reference sequence database (E-value threshold: 1e–10), ultimately yielding complete viral genome sequences [11, 12].

### 2.4. Phylogenetic Analysis

Reference sequences representing different genotypes and subtypes of PRRSV were downloaded from the GenBank (National Center for Biotechnology Information, 2025) database. These sequences, together with those obtained in this study, were aligned using MAFFT (parameter:—auto) [13]. Low-quality alignment regions were removed using TrimAl (parameter: -automated1), followed by manual curation to ensure alignment accuracy [14]. The processed sequences were subjected to maximum likelihood (ML) phylogenetic analysis using IQ-TREE with the best substitution model automatically selected by ModelFinder and branch reliability assessed by 1000 ultrafast bootstrap replicates [15]. The resulting phylogenetic trees were visualized and annotated using FigTree (http://tree.bio.ed.ac.uk/ software/figtree/) software.

### 2.5. Transmission Analysis

To elucidate the lineage-specific transmission dynamics of PRRSV type II in China, we performed spatiotemporal analyses for sublineages 1.5, 1.8, and Lineage 3 using the Nextstrain toolkit [16]. A total of 160 ORF5 sequences with precise temporal and geographical annotations were retrieved from GenBank, comprising 20 sequences from sublineage 1.5, 108 from sublineage 1.8, and 32 from Lineage 3. These were integrated with 250 ORF5 sequences generated in this study, corresponding to the same lineages (L1.5 = 73, L1.8 = 164, L3 = 13). The combined dataset of 410 sequences was subjected to a time-calibrated phylogenetic analysis using TreeTime to infer the phylogenetic relationships and transmission histories, estimating the timing and locations of transmission events.

Sequences were initially aligned using the augur align command to ensure a consistent layout. A preliminary ML tree was constructed with IQ-TREE 2, employing ModelFinder to identify the optimal substitution model. This ML tree served as the input for TreeTime via Augur to generate a time-resolved phylogeny [15]. Geographic metadata was subsequently mapped onto the phylogeny using the augur traits command. Finally, TreeTime was utilized to jointly estimate the phylogenetic positions and ancestral locations of all internal nodes, enabling a detailed assessment of the spatiotemporal transmission patterns for each lineage. Detailed information for the reference sequences retrieved from GenBank for this analysis is provided in Supporting Information 1: Table S1.

### 2.6. Diversity Analysis

Genetic diversity analyses of Chinese PRRSV type II ORF5 sequences from 2024 to 2025 were performed using DnaSP 6.0 [17]. Sequences were classified by lineage (1.5, 1.8, 3, 5, and 8.7) and grouped by collection year (2024 and 2025). Various genetic diversity parameters were calculated for each lineage, including haplotype diversity (Hd), nucleotide diversity (Pi), number of polymorphic sites (*S*), total number of mutations (Eta), and average number of nucleotide differences (*k*). Sliding window analysis (window length: 100 bp, step size: 3 bp) was employed to identify variation hotspots within the ORF5 gene. Further differentiation analysis between years included calculation of fixed differences, private polymorphic sites, shared mutations, average number of nucleotide differences between populations, average nucleotide substitution rate (Dxy), and net nucleotide substitution rate (Da). Tajima's D test was applied to assess selection pressure and infer population dynamic changes. All analyses excluded sites with gaps to ensure result accuracy.

### 2.7. Antigenic Variation Analysis

To analyze the antigenic relationships between circulating field strains and vaccine strains, we obtained sequences of several commonly used vaccine strains and their parent strains from the NCBI database, including JXA1 (GenBank accession No.EF112445.1) and its vaccine strain JXA1-P80 (FJ548853.1), CH-1R (EU807840.1) and its vaccine strain CH-1a (AY032626.1), HuN4 (EF635006.1) and its vaccine strain HuN4-F112, TJ (EU860248.1) and its vaccine strain TJM-F92 (MN508255.1), GD (EU825724.1) and its vaccine strain GDr180, VR-2332 (AY150564.1) and its vaccine derivative RespPRRS MLV (AF066183.4), and JA142 (AY424271.1) and its vaccine strain Ingelvac_ATP (DQ988080.1). For the commercial vaccine strains HuN4-F112 and GDr180, their ORF5 gene sequences were extracted from their complete genomes, which were determined in this study. These sequences, together with 67 reference sequences and sequences obtained in this study, were subjected to multidimensional scaling (MDS) analysis using R. A genetic distance matrix was calculated using the K80 model. The first two MDS dimensions captured 49.6% of the total variation, effectively delineating clear clustering relationships among the different lineages. This was followed by MDS dimensionality reduction to visualize the distribution patterns and clustering relationships of different lineages and vaccine strains in multidimensional space, enabling assessment of the antigenic match between current vaccine strains and circulating field strains.

### 2.8. iSNV Analysis

To detect genetic diversity and quasispecies structure within PRRSV populations, iSNV analysis was performed on whole genome sequencing data. Cleaned sequencing reads were mapped to reference genomes using BWA-MEM, followed by sorting and duplicate marking using SAMtools [18, 19]. Variant sites were identified based on SAMtools mpileup results. A minimum coverage threshold of 10x and a variant frequency threshold of 1% were applied to minimize sequencing error impacts. For each variant site, information including genomic position, variant type, coverage depth, variant allele count, and variant frequency was recorded, and functional annotation was performed based on the corresponding gene region of each variant position.

### 2.9. Recombination Analysis

Recombination events and breakpoints in whole genome sequences were detected using seven methods (RDP, GENECONV, BootScan, MaxChi, Chimaera, SiScan, and 3Seq) implemented in the RDP4 software package [20]. A recombination event was considered reliable when detected by at least five methods with a *p*-value ≤ 0.001. SimPlot (https://sray.med.som.jhmi.edu/SCRoftware/SimPlot/) analysis (window size: 500 bp, step size: 20 bp) was used for visual verification of recombination regions, and separate phylogenetic trees were constructed for regions on either side of recombination breakpoints to further confirm recombination events through topological changes. The recombination analysis results were used to identify recombination hotspots in the PRRSV type II genome and patterns of genetic exchange between different lineages.

## 3. Results

### 3.1. Sample Collection and PRRSV Type II Lineage Distribution

**Figure 1:**
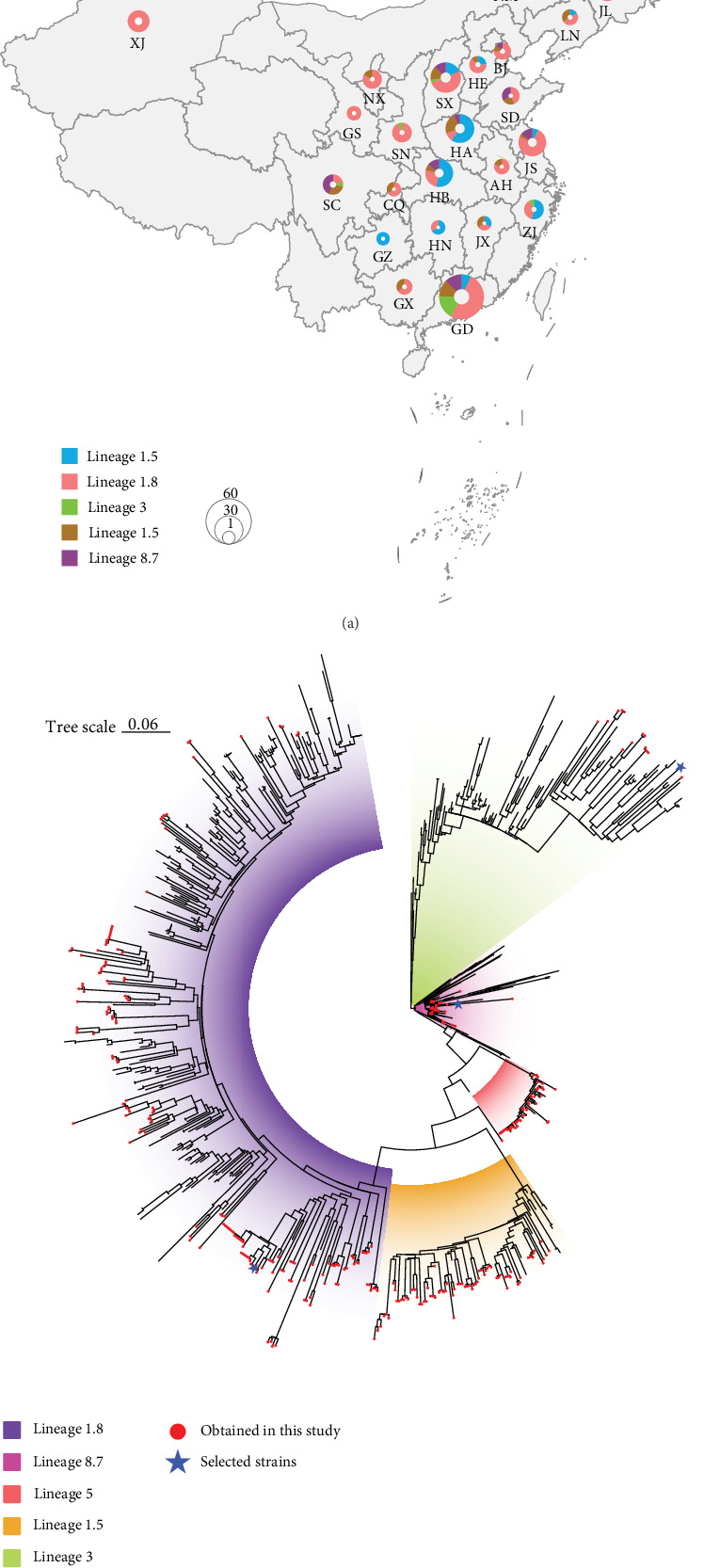
Sample collection and phylogenetic analysis. (a) Circle size in each province represents the number of samples, with colors denoting different lineages. (b) ML tree of PRRSV genotype II in China. Red circles indicate sequences obtained in this study, and blue asterisks denote full-genome sequences acquired.

During 2024–2025, we collected 328 samples from 27 provinces in China from pigs exhibiting typical PRRS symptoms. The geographical distribution of samples is shown in Figure 1a, with Guangdong (*n* = 59, 18.0%), Shanxi (*n* = 33, 10.1%), Henan (*n* = 30, 9.0%), Hubei (*n* = 29, 8.8%), and Jiangsu (*n* = 28, 8.5%) being the five provinces with the highest number of samples, accounting for 54.6% of the total samples.

Phylogenetic analysis revealed that PRRSV type II circulating in China could be classified into five major Lineages: Lineage 1.5, Lineage 1.8, Lineage 3, Lineage 5, and Lineage 8.7 (Figure 1b). To further understand the temporal dynamics of these lineages, we analyzed monthly PRRSV detection data from November 2024 to March 2025 (Table 1). Over this period, a total of 328 positive samples were recorded, with Lineage 1.8 being the most prevalent (159 cases, 48.5%) and Lineage 3 the least (14 cases, 4.0%). Detections peaked in February 2025 with 100 cases, driven primarily by Lineage 1.8 (48 cases) and Lineage 1.5 (29 cases). Lineage 1 (including Lineage 1.5 and Lineage 1.8) dominated most regions across the country, while Lineage 3, Lineage 5, and Lineage 8.7 showed relatively limited distribution. Notably, coexistence of multiple lineages was observed in provinces, such as Guangdong and Shanxi (Figure 1a), suggesting these regions might be important sites for PRRSV genetic exchange. Phylogenetic trees (Figure 1b) demonstrated that Lineage 1.5 and Lineage 1.8 formed relatively independent evolutionary branches, while Lineage 3 strains exhibited a high degree of genetic diversity, showing a more dispersed distribution pattern in the phylogenetic tree.

We selected three representative strains from Lineages 1.8, 3, and 8.7 for complete-genome sequencing. These strains were chosen based on their distinct phylogenetic positions and epidemiological relevance. Specifically, our selection criteria prioritized: (1) the predominant and rapidly spreading lineage (Lineage 1.8), (2) a lineage with close ties to commercial vaccines (Lineage 8.7), and (3) a genetically distinct and regionally significant lineage (Lineage 3). The Lineage 1.8 strain (P24110708wuhan241107) was chosen as it represents the rapidly spreading NADC30-like sublineage, which has been associated with recent outbreaks in China. The Lineage 8.7 strain (R24121302heyuan241213) was selected for a close phylogenetic relationship with vaccine strains, such as HuN4-vaccine, GDR180-vaccine, and TJM92-vaccine, making it crucial for understanding vaccine-related evolutionary pressures. The Lineage 3 strain (P25012805heyuan250128) exhibits low nucleotide similarity (90.9%) with other ORF5 strains and forms a relatively independent branch within Lineage 3, suggesting it may represent a distinct evolutionary trajectory (Figure 1b). Whole-genome sequencing of these strains achieved an average depth of coverage exceeding 50x, providing a reliable data foundation for subsequent analyses (Supporting Information 2: Figure S1).

### 3.2. Transmission Dynamics Analysis of PRRSV Type II

**Figure 2:**
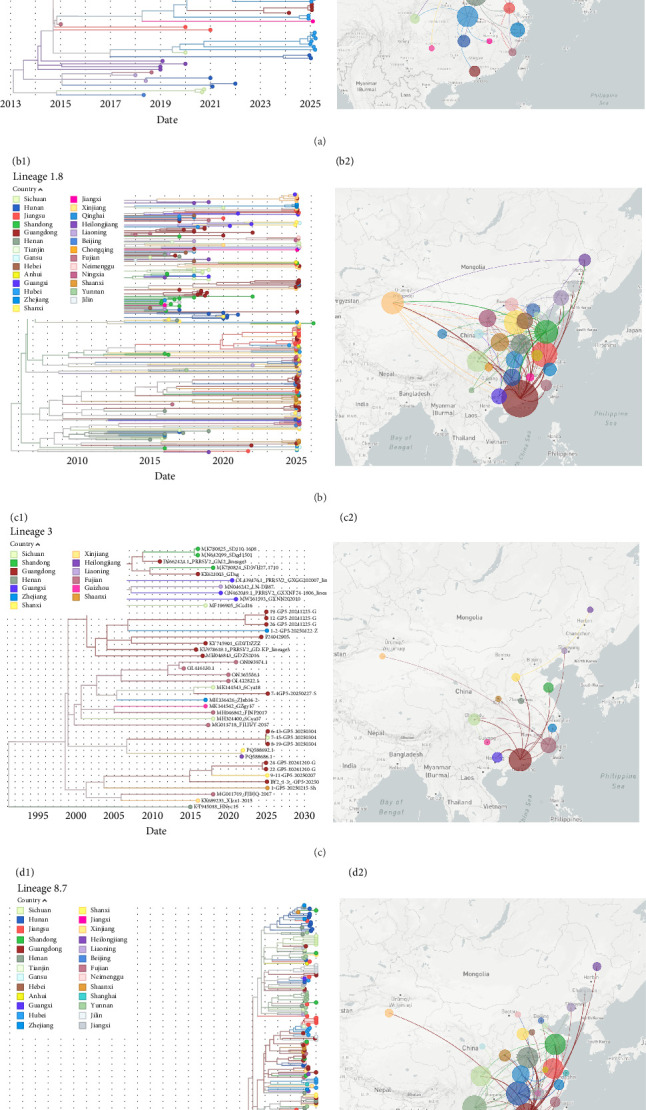
Transmission analysis. (a1, b1, c1, d1): phylogenetic tree constructed by maximum likelihood method based on ORF5 using Nextstrain, with different provinces color-annotated on branches. (a2, b2, c2, d2): Phylogeographic reconstruction of PRRSV ORF5, where circle size represents sample quantity and colors correspond to ancestral nodes.

Phylogeographic analyses based on Nextstrain revealed clear differences in the spatial and temporal transmission dynamics among the various PRRSV type II lineages circulating in China. Overall, Lineages 1.5 and 1.8 exhibited extensive geographic dissemination over the past decade, while Lineage 3 and Lineage 8.7 displayed more regionalized transmission patterns (Figure 2).

Specifically, Lineage 1.5 originated in Heilongjiang around 2015 and subsequently expanded its range, with major transmission routes extending toward central and eastern provinces. By approximately 2025, this Lineage was predominantly distributed in Henan and surrounding provinces, including Hubei, Zhejiang, Shanxi, and Hebei, reflecting a clear shift in its epidemic center over time. In contrast, Lineage 1.8 was first reported in China around 2012 [21], and its spread was most notable between 2015 and 2020 in Guangdong, Henan, and Sichuan. By 2025, transmission had further extended primarily to provinces neighboring Guangdong and Henan, such as Hunan, Hubei, Hebei, Jiangsu, and Shaanxi, indicating ongoing outward dissemination from these key regions. Lineage 3 showed a distinct, localized transmission profile, originating in Guangdong around 2007 and remaining largely confined to the southern coastal region. From 2020 to 2025, its spread was mainly restricted to Guangdong, Guangxi, and Fujian, highlighting its limited cross-regional transmission. Additionally, Lineage 8.7 was found to be mainly concentrated in Guangdong, Henan, Sichuan, and Hunan between 2015 and 2025, with Guangdong serving as a principal epicenter for local and neighboring province transmission.

Collectively, these findings demonstrate the divergent transmission trajectories among lineages and underscore the importance of regional surveillance and tailored intervention strategies to contain PRRSV dissemination in China.

### 3.3. Genetic Diversity Characteristics of PRRSV Type II

**Figure 3:**
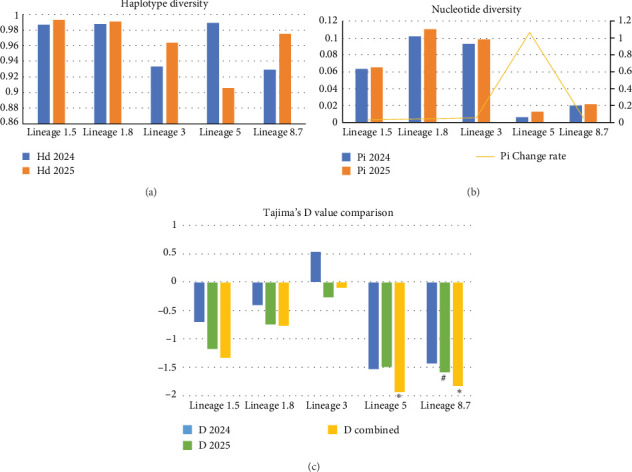
Genetic diversity analysis of Type II PRRSV ORF5 gene across five major lineages in China (2024–2025). (a) Haplotype diversity (Hd) analysis of lineages 1.5, 1.8, 3, 5, and 8.7. (b) Nucleotide diversity (Pi) analysis. (c) Tajima's D test results, well number, and asterisks indicate statistical significance (*p* < 0.05).

Diversity analysis of ORF5 gene sequences from the five major lineages is shown in Figure 3. Hd analysis (Figure 3a) showed that all lineages exhibited high Hd values (Hd index; ranging from 0.905 to 0.993), indicating a high degree of genetic variation among circulating PRRSV in China. Most lineages maintained stable or slightly increased Hd between 2024 and 2025, while the Hd value of Lineage 5 decreased from 0.989 in 2024–0.905 in 2025, showing a unique evolutionary pattern.

Nucleotide diversity analysis (Figure 3b) revealed significant differences among different lineages. Lineage 1.8 exhibited the highest nucleotide diversity (0.10478 in 2024 and 0.11064 in 2025), followed by Lineage 3 (0.09322 in 2024 and 0.09839 in 2025) and Lineage 1.5 (0.06343 in 2024 and 0.06548 in 2025). In contrast, Lineage 5 and Lineage 8.7 showed significantly lower nucleotide diversity (0.00620-0.01281 and 0.02024-0.02128, respectively). Notably, all lineages exhibited increasing trends in nucleotide diversity between 2024 and 2025, with Lineage 5 showing the most significant growth (an increase of 106.6%), as indicated by the yellow line in Figure 3b.

Tajima's D test results (Figure 3c) showed negative values for all lineages across different years and in combined samples, except for Lineage 3 samples from 2024 (*D* = 0.53280). Notably, the combined samples for Lineage 5 (*D* = −1.93258, *p*  < 0.05) and Lineage 8.7 (D = −1.82718, *p*  < 0.05) reached statistical significance. These negative values suggest that these viral populations may have experienced recent population expansion or directional selection.

Sliding window analysis (Supporting Information 2: Figure S2) further revealed variation hotspots within the ORF5 gene, generally showing higher variation at the 5′ and 3′ ends compared to the middle region, consistent with the structural characteristics of the GP5 protein. The variation hotspot regions across lineages remained relatively stable between 2024 and 2025, although the magnitude of variation increased, particularly in Lineage 5.

### 3.4. Antigenic Variation Analysis

**Figure 4:**
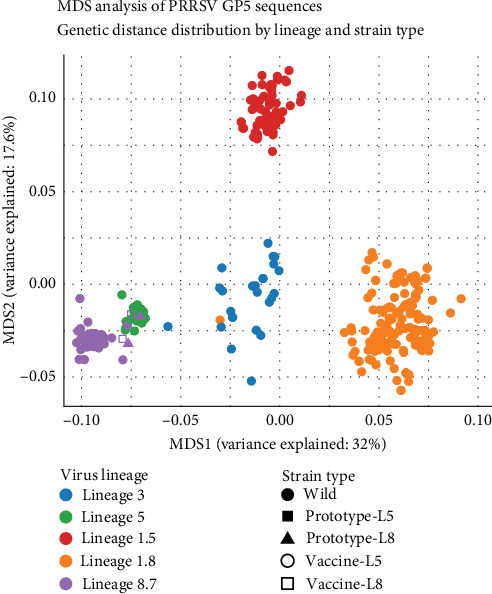
MDS clustering analysis of vaccine strains, prototype strains, and field samples. Different colors represent different lineages, and shapes distinguish vaccine strains, prototype strains, and field strains.

MDS analysis results (Figure 4) revealed the distribution patterns of different lineages in antigenic space. The results showed that different lineages formed distinct clusters, suggesting they may possess different antigenic properties. Among them, Lineage 1.5 (red) formed tight clustering in multidimensional space, indicating high antigenic homogeneity within this lineage.

Notably, Lineage 5 (green) and Lineage 8.7 (purple) were relatively close to the analyzed vaccine strains in multidimensional space, suggesting potential antigenic similarity between existing vaccine strains and these two lineages. In contrast, Lineage 3 (blue) and Lineage 1.8 (orange) were distinctly separated from Lineage 5 and Lineage 8.7 on the MDS plot, implying significant antigenic differences between them. Furthermore, Lineage 3 strains showed relatively dispersed distribution in space, suggesting potentially high antigenic diversity within this lineage.

### 3.5. Recombination Analysis

**Figure 5:**
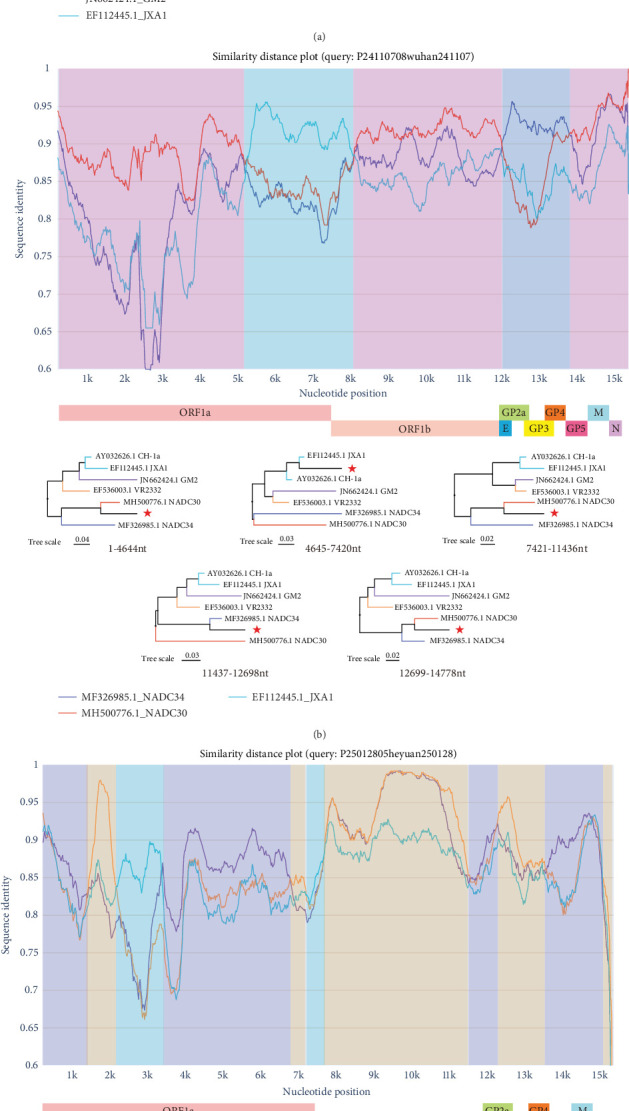
Recombination analysis. (a) Recombination analysis of strain R24121302heyuan241213. Different colors represent distinct strains, and the red star indicates R24121302heyuan241213. (b) Recombination analysis of strain P24110708wuhan241107. Different colors represent distinct strains, and the red star indicates P24110708wuhan241107. (c) Recombination analysis of strain P25012805heyuan250128. Different colors represent distinct strains, and the red star indicates P25012805heyuan250128.

To investigate the genomic underpinnings of viral evolution, we conducted an in-depth recombination analysis on the three representative whole genomes. This case study revealed significant and complex gene recombination events in all three strains, highlighting recombination as a crucial evolutionary mechanism. Phylogenetic analyses revealed that while each genome displayed a unique recombination pattern, certain common features were also observed (Figure 5a–c).

All three samples utilized HP-PRRSV JXA1 as a major parental strain in recombination events, yet each exhibited distinct recombination patterns and secondary parental strain preferences. The Lineage 8.7 sample (R24121302heyuan241213) predominantly employed JXA1 as the major parent, with recombination involving the GM2 strain detected in the 11681–13040nt region (Figure 5a). The Lineage 1.8 sample (P24110708wuhan241107) exhibited recombination with the JXA1 strain in the 4645–7420 region and with the NADC34 strain in the 12699–14778 region (Figure 5b).

The Lineage 3 sample (P25012805heyuan250128) displayed the most complex recombination pattern, featuring alternating recombination with VR2332 and JXA1 strains across multiple genomic regions (Figure 5c).

A key observation from this case study was that the ORF2-ORF3 region (approximately 11600–13500nt) appeared to be a potential recombination hotspot, as recombination events were detected in this region across all three distinct genomes. Notably, this region encodes viral envelope glycoproteins involved in host cell receptor binding and immune evasion. Additionally, multiple fragments within the ORF1b region exhibited high recombination frequency. While these findings are based on a limited number of genomes, the cumulative effect of such recombination events could potentially generate recombinant viruses with novel antigenic properties or enhanced virulence, challenging the protective efficacy of current vaccines.

### 3.6. iSNV Analysis

**Figure 6:**
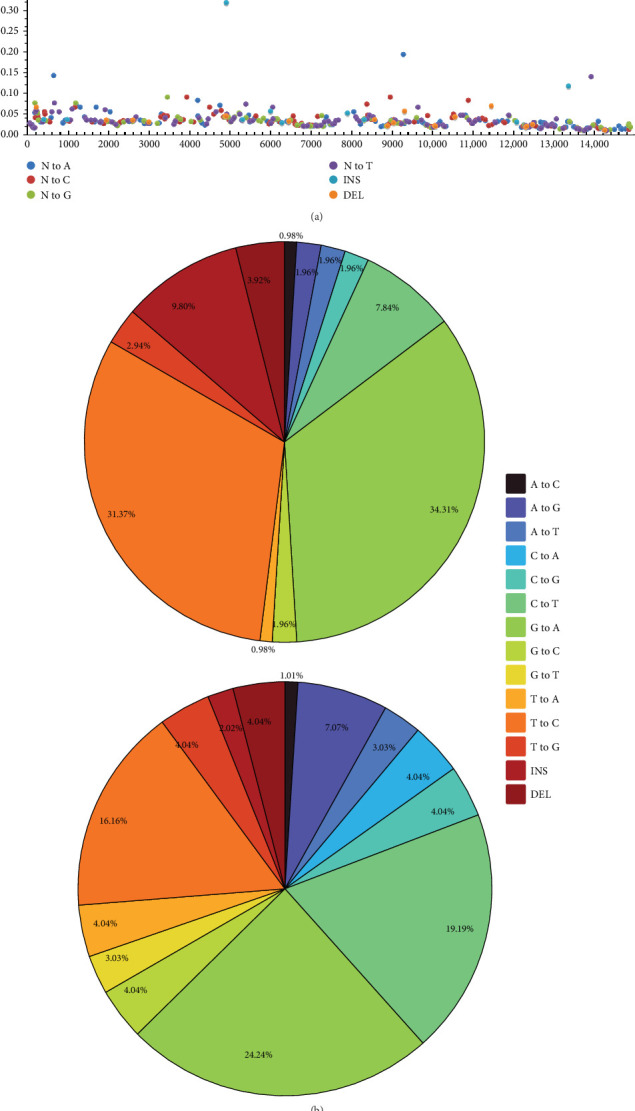
iSNV Analysis. (a) Different colors represent various mutation types (insertions/deletions). (b) Relative frequency plot of mutation types. (c) Number of mutations at different protein positions. (d) Mutation counts in structural and nonstructural protein regions.

To understand the variation characteristics of PRRSV type II within hosts, we conducted iSNV analysis on samples representing two different lineages, as shown in Figure 6. The variant site distribution plot (Figure 6a) showed that the Lineage 8.7 sample (R24121302heyuan241213) contained significantly more variant sites (512) than the Lineage 1.8 sample (P24110708wuhan241107, 120). Base substitution type analysis (Figure 6b) indicated that transitions (G↔A) predominated in both lineages, but with lineage-specific distribution patterns. The strain P24110708wuhan241107 showed a higher proportion of T→C transitions (31.37%) compared to the strain R24121302heyuan241213 (16.16%).

The iSNV distribution in the structural protein (SP) regions (Figure 6c) exhibited similar region specific patterns. Both samples had a relatively high number of variations in the GP2, GP4, and GP5 regions. By contrast, fewer variations were observed in the M protein (GP6) and N protein (GP7) regions, suggesting that these regions might be subject to stronger functional constraints. Variation distribution based on genomic regions (Figure 6d) showed that in both strains, the number of variant sites in non-structural protein regions (NSP) significantly exceeded those in SP regions. The NSP:SP variation ratio was 3.4:1 (379:111) in the Lineage 8.7 sample and 3.9:1 (86:22) in the Lineage 1.8 sample.

## 4. Discussion

This molecular epidemiological investigation reveals the cocirculation of five major PRRSV Type II Lineages (1.5, 1.8, 3, 5, and 8.7) across China during 2024–2025, with Lineage 1.8 predominating (48.5%). While Lineages 1.5 and 1.8 show widespread distribution, Lineages 3, 5, and 8.7 display more restricted geographical patterns. Provinces like Guangdong and Shanxi exhibit multiple cocirculating lineages, potentially facilitating genetic exchange. Our analyses confirmed significant genetic diversity across lineages, distinct antigenic relationships among circulating strains relative to vaccine strains, extensive recombination with the ORF2-ORF3 region as a major hotspot, and lineage-specific patterns of intrahost variation.

The predominance of Lineage 1.8 observed in our study aligns with recent findings by Zhang et al. [4], who documented the rapid expansion of NADC30-like strains (Lineage 1.8) in China. Their research similarly identified a shift in dominant circulating strains from classic Lineage 8.7 variants toward Lineage 1.8, attributing this change to the strain's enhanced transmissibility and potential immune escape capabilities. The continued prevalence of Lineage 1.8 strains in our 2024–2025 sampling suggests this lineage has maintained its competitive advantage, potentially through ongoing evolution and adaptation. Our transmission dynamics analysis revealed distinct spatiotemporal patterns for different lineages, with Lineages 1.5 and 1.8 showing extensive geographical dissemination compared to the more regionally confined Lineage 3. These patterns likely reflect differences in viral fitness, host adaptation, and potentially human-mediated transportation factors. Similar heterogeneous transmission patterns were documented by Yuan et al. [22], who identified certain PRRSV lineages with broader geographical spread capabilities than others in their 3-year surveillance study. The restricted distribution of Lineage 3 primarily in southern China (Guangdong, Guangxi, and Fujian) suggests potential ecological or other factors limiting its spread to northern regions, an observation that warrants further investigation.

The high genetic diversity observed across all lineages, particularly within Lineage 1.8, indicates ongoing evolutionary processes driving PRRSV adaptation. The negative Tajima's D values detected for most lineages suggest recent population expansion or purifying selection. The exceptionally high nucleotide diversity in Lineage 1.8 (Pi = 0.11064 in 2025) indicates substantial genetic heterogeneity within this lineage, which may contribute to its epidemiological success through enhanced adaptability to varying host and environmental pressures. Interestingly, the significant increase in nucleotide diversity for Lineage 5 between 2024 and 2025 (106.6% growth) suggests this lineage may be undergoing rapid evolutionary changes that warrant close monitoring.

Recombination has long been recognized as a major driver of PRRSV evolution, and our findings are consistent with its continued importance. The observation in our representative strains that the ORF2-ORF3 region as a recombination hotspot corroborates previous findings by Cui et al. [23], who similarly identified this region as a frequent recombination site. The complex recombination pattern observed in our Lineage 3 representative genome, involving alternating segments derived from VR2332 and JXA1 strains, demonstrates the intricate evolutionary history of circulating PRRSV strains. These recombination events likely contribute to the observed genetic diversity and may facilitate adaptation to changing selective pressures, including vaccine-induced immunity. Collectively, our recombination analyses reveal three distinct recombination patterns in the three representative genomes examined.. The first pattern, observed in the representative genome from Lineage 8.7, represents a relatively simple recombination event with a single crossover point primarily in the ORF2-ORF3 region. The second pattern, detected in the representative genome from Lineage 1.8, demonstrates a dual-recombination scenario with separate crossover events in both ORF1b and ORF2-ORF5 regions. The third and most complex pattern, identified in the representative genome from Lineage 3, exhibits multiple alternating recombination events throughout the genome, creating a mosaic structure derived from different parental strains. These diverse recombination patterns suggest that different viral strains may utilize distinct evolutionary strategies, potentially influencing viral fitness and pathogenicity. Furthermore, the consistent involvement of HP-PRRSV vaccine strain JXA1 as a major parental strain in all three genomes we examined suggests its genetic elements may provide selective advantages for circulating viruses; a finding that raises concerns about the field safety of current live vaccines and warranting further investigation into the functional consequences of these recombination events.

Our intra-host SNV analysis revealed striking differences in viral population diversity between lineages, with the Lineage 8.7 sample harboring significantly more variant sites than the Lineage 1.8 sample. The predominance of transitions over transversions in our data is consistent with general patterns of RNA virus evolution, while the higher proportion of structural variations in the Lineage 1.8 sample may reflect different evolutionary strategies between lineages. The significantly higher variation in GP2, GP3, and GP5 regions compared to the more conserved M and N proteins highlights the differential selective pressures acting on different viral proteins, with structural glycoproteins typically exhibiting greater variability due to immune selection pressures.

From a disease control perspective, the results of our antigenic variation analysis are particularly concerning. Our computational analysis, based on genetic sequences, predicts a potential antigenic divergence between the predominant circulating lineages (1.8 and 3) and current vaccine strains. Although this finding requires definitive confirmation through serological assays, it suggests that the efficacy of current vaccines may be compromised, potentially contributing to ongoing PRRSV persistence and evolution despite vaccination efforts. Similar concerns were raised by Wei et al. [24], who documented reduced cross-protection between commercial vaccines and emerging PRRSV variants. In contrast, the closer antigenic relationship between vaccine strains and Lineages 5 and 8.7 suggests potentially better vaccine coverage against these less prevalent lineages.

The study's limitations include an uneven geographical sample distribution, which may have biased the estimations of lineage prevalence and transmission hubs. Furthermore, the in-depth genomic analyses are based on three representative strains; therefore, conclusions regarding recombination and evolutionary patterns should be considered preliminary hypotheses that require validation with larger datasets.

In conclusion, our findings highlight the dynamic and complex evolutionary landscape of PRRSV Type II in China, characterized by multiple cocirculating lineages with distinct genetic, geographic, and antigenic profiles. The predominance of Lineage 1.8, coupled with its high genetic diversity and antigenic divergence from current vaccine strains, raises concerns for PRRS control efforts. Continued surveillance and timely updating of preventive strategies, including vaccine formulations, will be essential to address the challenges posed by the evolving PRRSV population. Further research into the biological significance of the observed genetic and antigenic variations, particularly their impact on virulence and immune evasion is warranted to inform more effective control strategies.

## Figures and Tables

**Table 1:** Monthly PRRSV detection statistics.

Month	Month total	Lineage 1.5	Lineage 1.8	Lineage 3	Lineage 5	Lineage 8.7
2024–11	18	6	5	0	4	3
2024–12	68	11	32	5	16	4
2025–01	87	21	45	3	6	12
2025–02	100	29	48	3	7	13
2025–03	55	9	29	3	9	5
Total	328	76	159	14	42	37

## Data Availability

The data presented in this study are available upon request from the corresponding author. The data are not publicly available due to privacy protection.

## Supporting Information

Supporting Information 1 The information of the viral strain sequences employed in this study.

Supporting Information 2 Figure S1 Annotation and Depth of Complete-Genome Sequences. The blue line represents the depth at each position in the genome, and the content labeled by the maroon arrows below denotes the positions of different proteins on the genome. Figure S2 Sliding Window Analysis Results. (a) Nucleotide diversity (Pi) sliding window analysis by lineage (2024–2025). (b) Tajima's D test results sliding window analysis by lineage (2024–2025). (c) Dxy (between-lineage nucleotide divergence) and Da (within-lineage average nucleotide difference) sliding window analysis by lineage (2024–2025).
