# Correction to “Molecular Epidemiology, Lineage Evolutionary Dynamics, and Antigenic Variation Analysis of Type II PRRSV in China During 2024–2025”

**DOI:** 10.1155/tbed/9840749

**Published:** 2026-04-17

D. Zhu, G. Liu, H. Li, et al., “Molecular Epidemiology, Lineage Evolutionary Dynamics, and Antigenic Variation Analysis of Type II PRRSV in China During 2024–2025,” *Transboundary and Emerging Diseases* 2025, no. 1 (2025): 1–18, https://doi.org/10.1155/tbed/2054759.

In the article titled “Molecular Epidemiology, Lineage Evolutionary Dynamics, and Antigenic Variation Analysis of Type II PRRSV in China During 2024–2025” there is an error in Figure 1a. The brown coloured marker is incorrectly labelled as ‘Lineage 1.5’, instead of ‘Lineage 5’, which was not in keeping with the rest of the article. This occurred due to a labelling error.

Figure 1a should be corrected as follows:

**Figure 1:**
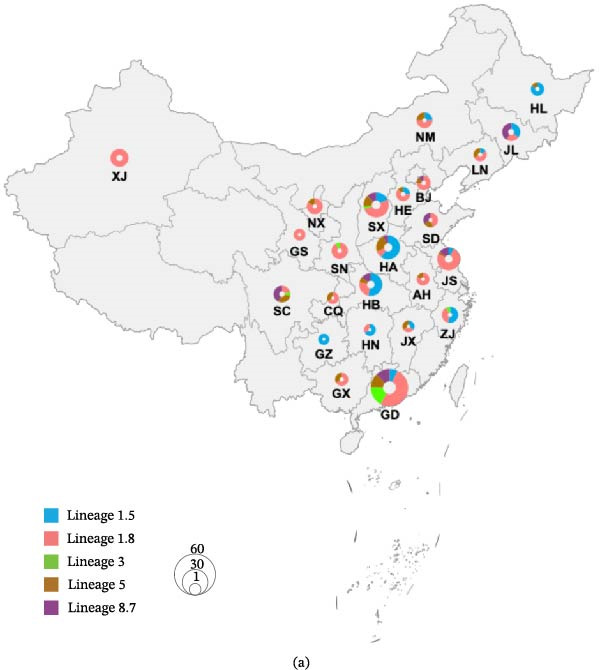
Sample collection and phylogenetic analysis. (a) Circle size in each province represents the number of samples, with colors denoting different lineages.

We apologize for this error.

