# Supplementary material for: Molecular Epidemiology, Lineage Evolutionary Dynamics, and Antigenic Variation Analysis of Type II PRRSV in China During 2024–2025
Source: Transbound Emerg Dis. 2025 Sep 27;2025:2054759. doi: 10.1155/tbed/2054759 (PMC12496146; doi:10.1155/tbed/2054759)

**Fig S1 Annotation and Depth of Complete-Genome Sequences**

The blue line represents the depth at each position in the genome, and the content labeled by the maroon arrows below denotes the positions of different proteins on the genome.


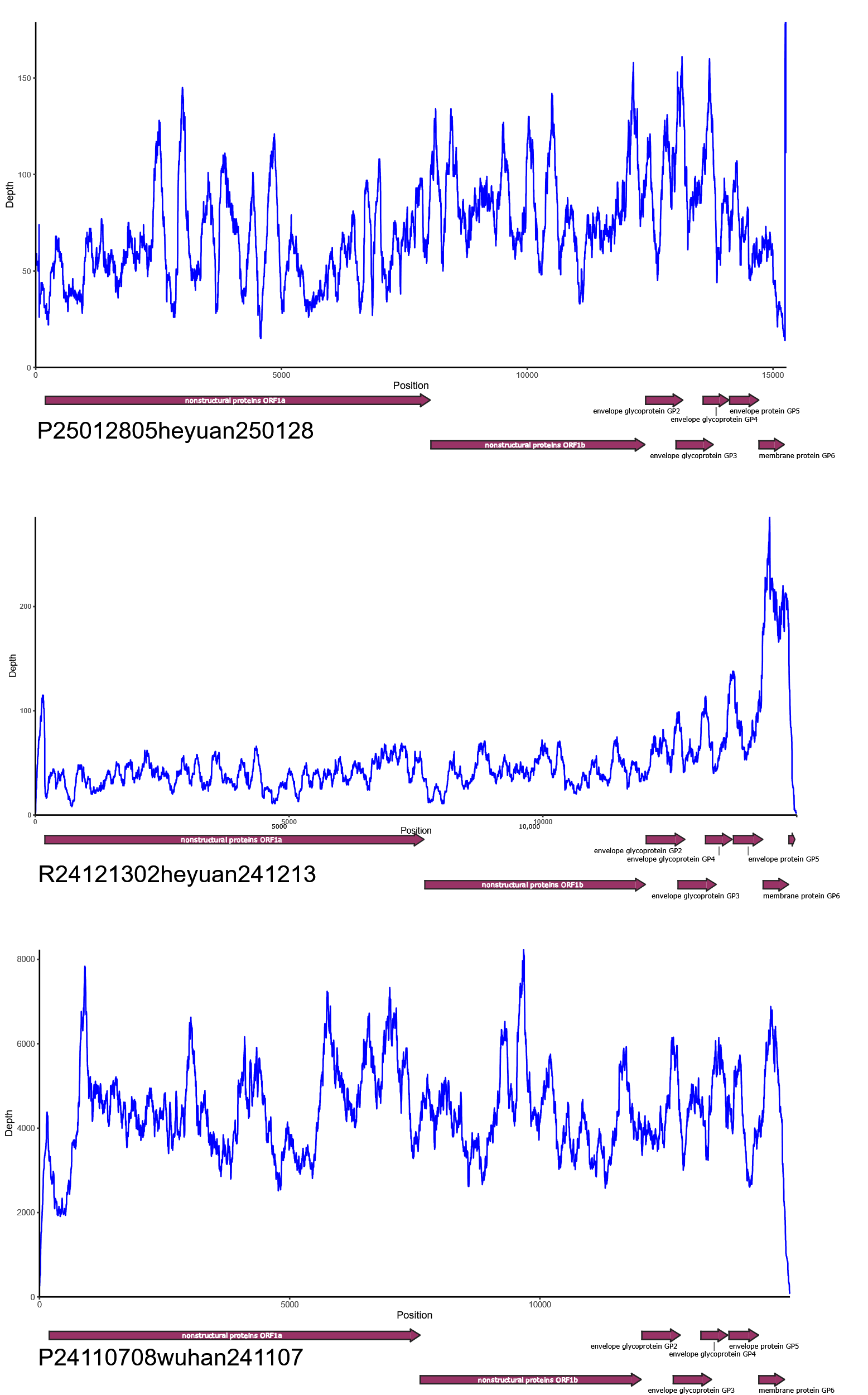


**FigS2 Sliding Window Analysis Results**

a Nucleotide Diversity (Pi) Sliding Window Analysis by Lineage (2024–2025).

b Tajima’s D Test Results Sliding Window Analysis by lineage (2024–2025).

c Dxy（between-lineage nucleotide divergence）and da（within-lineage average nucleotide difference）Sliding Window Analysis by lineage (2024–2025).


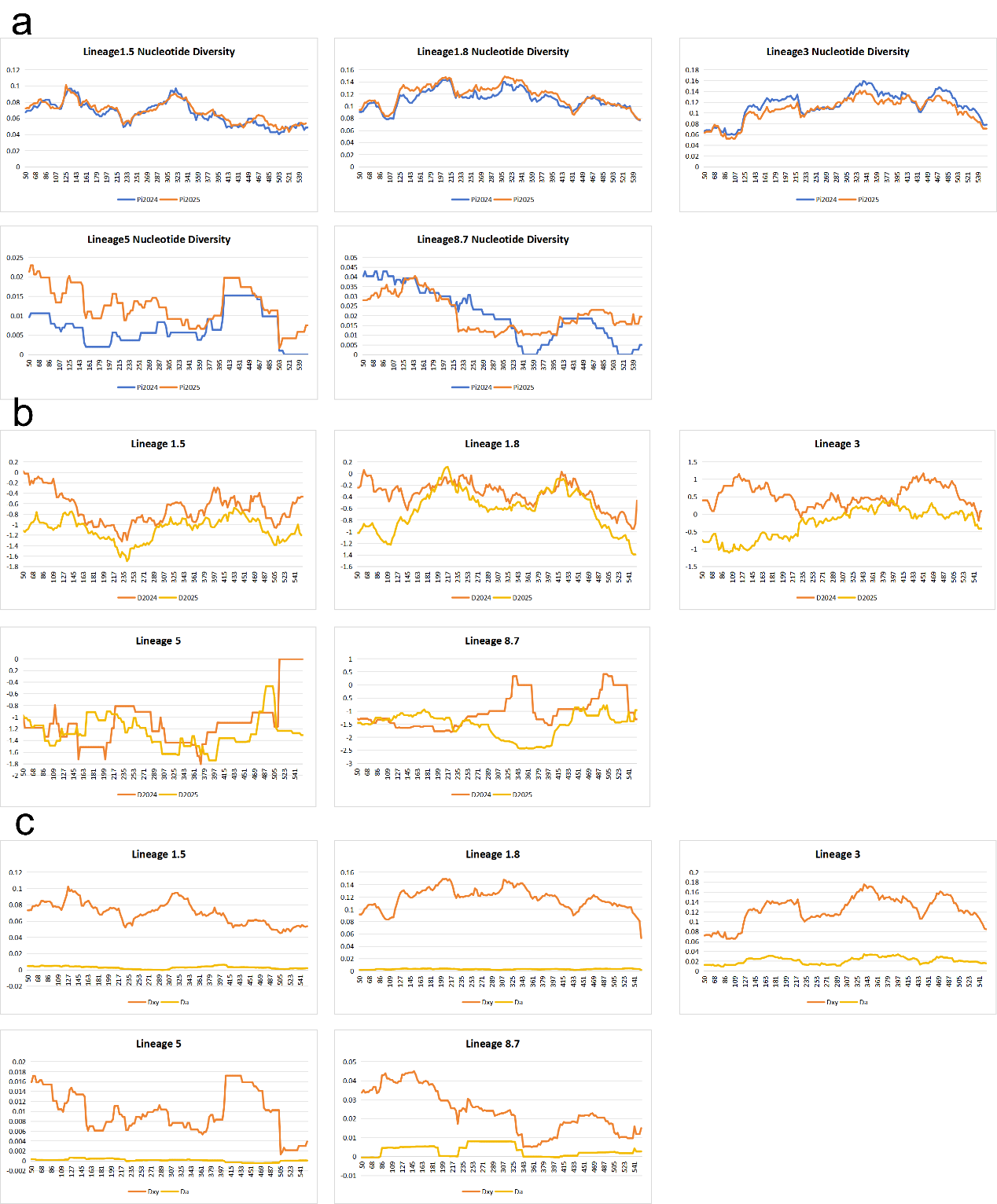

Supplement: Supporting Information 2 — Figure S1 Annotation and Depth of Complete-Genome Sequences. The blue line represents the depth at each position in the genome, and the content labeled by the maroon arrows below denotes the positions of different proteins on the genome. Figure S2 Sliding Window Analysis Results. (a) Nucleotide diversity (Pi) sliding window analysis by lineage (2024–2025). (b) Tajima's D test results sliding window analysis by lineage (2024–2025). (c) Dxy (between-lineage nucleotide divergence) and Da (within-lineage average nucleotide difference) sliding window analysis by lineage (2024–2025). [file 2054759.f2.docx]
